# Polarizable Water Model for the Coarse-Grained MARTINI Force Field

**DOI:** 10.1371/journal.pcbi.1000810

**Published:** 2010-06-10

**Authors:** Semen O. Yesylevskyy, Lars V. Schäfer, Durba Sengupta, Siewert J. Marrink

**Affiliations:** 1 Department of Physics of Biological Systems, Institute of Physics, National Academy of Sciences of Ukraine, Kiev, Ukraine; 2 Groningen Biomolecular Sciences and Biotechnology Institute & Zernike Institute for Advanced Materials, University of Groningen, Groningen, The Netherlands; Stanford University, United States of America

## Abstract

Coarse-grained (CG) simulations have become an essential tool to study a large variety of biomolecular processes, exploring temporal and spatial scales inaccessible to traditional models of atomistic resolution. One of the major simplifications of CG models is the representation of the solvent, which is either implicit or modeled explicitly as a van der Waals particle. The effect of polarization, and thus a proper screening of interactions depending on the local environment, is absent. Given the important role of water as a ubiquitous solvent in biological systems, its treatment is crucial to the properties derived from simulation studies. Here, we parameterize a polarizable coarse-grained water model to be used in combination with the CG MARTINI force field. Using a three-bead model to represent four water molecules, we show that the orientational polarizability of real water can be effectively accounted for. This has the consequence that the dielectric screening of bulk water is reproduced. At the same time, we parameterized our new water model such that bulk water density and oil/water partitioning data remain at the same level of accuracy as for the standard MARTINI force field. We apply the new model to two cases for which current CG force fields are inadequate. First, we address the transport of ions across a lipid membrane. The computed potential of mean force shows that the ions now naturally feel the change in dielectric medium when moving from the high dielectric aqueous phase toward the low dielectric membrane interior. In the second application we consider the electroporation process of both an oil slab and a lipid bilayer. The electrostatic field drives the formation of water filled pores in both cases, following a similar mechanism as seen with atomistically detailed models.

## Author Summary

Many biomolecular processes involve charged species moving between regions of high polarity, such as the water phase, and regions of lower polarity, such as the lipid membrane. Due to the change in electrostatic screening between these two environments, the strength of the interactions between the moving charge and the surrounding molecules also changes. This has important consequences for the way biological activity is controlled. To help understand the forces driving the movement of biomolecules, we developed a computational model which is capable of describing these processes at near-atomic detail. To do so efficiently, we use a coarse-grained description of the molecules, in which some of the atomistic detail is averaged out. To capture the inhomogeneous nature of the dielectric response, we re-introduce some detail in the water model; the new model effectively mimics the orientational polarizability of real water molecules, and screens electrostatic interactions realistically. This enables the study of a number of important biological processes that were hitherto considered challenging for coarse-grained models, such as the permeation of ions across a lipid membrane and the rupture of membranes due to an electrostatic field, at relatively low computational cost.

## Introduction

Since the first introduction of physics-based coarse-grained (CG) models in computational biology [1], CG models have become increasingly popular in the simulation of complex biological systems [2]. They significantly reduce the computational complexity in comparison to all-atom (AA) models and allow sampling over much longer time scales and of larger system sizes. One of the most widely applied CG models is the MARTINI force field [3]. The MARTINI model was initially developed for lipid systems [4] and has recently been extended for proteins [5] and carbohydrates [6]. In general a four-to-one mapping is used in MARTINI, which means that on average four atoms and associated hydrogens are represented by one CG bead. The CG particles interact with the other CG particles in the system by means of Lennard-Jones (LJ) interactions; in addition charged groups (e.g. ions, lipid head groups, charged amino acid side chains) interact via a Coulombic energy function. Water is treated explicitly, at the same level of coarse-graining as all other molecules implying that four water molecules are combined into a single coarse-grained bead.

MARTINI water beads, just as many other CG water models, do not bear charges and, consequently, are blind to electrostatic fields and polarization effects. To compensate for the neglect of explicit polarization, screening of electrostatic interactions is done implicitly, assuming a uniform relative dielectric constant. While this is a reasonable approximation for bulk water, problems arise at the interfaces between water and other phases and in the vicinity of charged particles. Because of the implicit screening, the interaction strength of polar substances is underestimated in non-polarizable solvents. Correct modeling of the partitioning of polar and charged compounds into a low dielectric medium, e.g. a lipid bilayer, has proven a big challenge for CG models in general [7]. Applications involving the formation of polar/charged complexes in a non-polar environment are especially prone to be affected. A potential solution is to make the interaction potentials dependent on the local environment (see e.g. [8]), especially useful in solvent free approaches. With explicit solvent particles present, more flexibility is achieved with a polarizable water model.

Attempts to include the effect of polarization in simplified water models date already back to the early days of biomolecular modeling. Notably the development of the soft sphere dipole model is worth mentioning [9], [10]. In this model, water molecules are represented by point dipoles that can reorient in response to the electrostatic field of an embedded (macro)molecule. Recently, induced dipoles were also added to a CG solvent model, and made compatible with a CG protein force field [11]. The polarizability challenge also stands in all-atom (AA) force fields at a more fine-grained level. The AA force fields lack electronic polarizability, which has proven to be a significant drawback in simulations of ions and highly polarizable systems [12], [13]. Several approaches to develop polarizable AA force fields, such as the inducible point dipole model [14], the model with Drude oscillators [15], [16], the fluctuating charge model [17] and the multipole expansion model [18] exist. The general idea of all these methods is to introduce a fluctuating dipole to each polarizable particle, which responds to the local electric field in the vicinity of this particle.

In this work, we introduce orientational polarizability to the water beads of the MARTINI force field using an approach similar to that of the Drude oscillator [15], [16]. The resulting polarizable CG water model, in combination with the MARTINI force field, allows modeling the interaction of water with charged particles in a more realistic way. In the parameterization of the polarizable water model the following three criteria were used: i) The dielectric constant of bulk polarizable water should be sufficiently close to the value in real water; ii) The particle density of the polarizable water should be close to the particle density of the water in standard MARTINI; iii) The reproduction of partitioning free energies between water and organic solvents for a large variety of small compounds, one of the corner stones of the MARTINI model, should remain unaffected.

The rest of this paper is organized as follows. In the next section, we first describe the details of the model, and the way we set out to parameterize it. This is followed by the Results section in which we explore the parameter space and arrive at the optimal parameter set, based on reproduction of the density and dielectric constant of bulk water, and the water/oil partitioning behavior of the MARTINI building blocks. We then test a number of properties of the new model, including the dynamical behavior of bulk water, the surface tension of the water/vapor interface, and structural properties of ionic solutions and of a lipid bilayer. We also look at the effect of long-range electrostatic interactions. Finally, two applications are shown which would not have been feasible with the standard MARTINI model, nor with most other CG models. The applications are a realistic description of the free energy of ion transport across a lipid bilayer, and the electroporation process of both an octane slab and a lipid bilayer. A discussion section about the limitations and prospects of the model concludes this paper.

## Methods

### Topology of the water model

**Figure 1:**
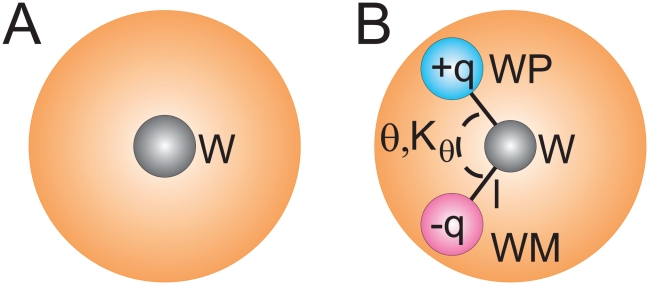
Water models used in the MARTINI force field. a) Standard model; b) polarizable model. Shaded orange spheres correspond to the van der Waals radii of the central particles W.

The polarizable CG water consists of three particles instead of one in the standard MARTINI force field (Fig. 1). The central particle W is neutral and interacts with other particles in the system by means of the Lennard-Jones interactions, just like the standard water particle. The additional particles WP and WM are bound to the central particle and carry a positive and negative charge of +*q* and −*q*, respectively. They interact with other particles via a Coulomb function only, and lack any LJ interactions. The bonds W-WP and W-WM are constrained to a distance *l*. The interactions between WP and WM particles inside the same CG water bead are excluded, thus these particles are “transparent” toward each other. As a result the charged particles can rotate around the W particle. The dipole momentum of the water bead depends on the position of the charged particles and can vary from zero (charged particles coincide) to 2*lq* (charged particles are at the maximal distance). A harmonic angle potential with equilibrium angle θ and force constant *K_θ_* is furthermore added to control the rotation of WP and WM particles and thus to adjust the distribution of the dipole momentum. The average dipole momentum of the water bead will depend on the charge distribution and is expected to be on average zero in an apolar environment, such as the interior of the lipid bilayer. In contrast, some non-zero average dipole will be observed in bulk water or in some other polar environment. The masses of the charged particles as well as of the central particle are set to 24 amu, totaling 72 amu (the mass of four real water molecules).

There are five adjustable parameters in the polarizable water particle: The charge *q*, the distance *l*, the angle parameters θ and *K*_θ_, and the atom type of the central particle W. The accessible range of the dipole momentum of the water bead is determined by both *l* and *q*; to restrict our parameter space we used *q* as the only adjustable parameter and fixed *l* at the value 0.14 nm. This distance is small enough to prevent the overlap of the charged particles of adjacent water beads (which could result in very large forces) and large enough to represent the instantaneous dipole of a cluster of four water molecules. Similarly, only *K*_θ_ was varied. The equilibrium angle was fixed at θ = 0 to ensure that the water bead in an apolar solvent has a vanishing dipole moment (recalling that one CG water bead effectively represents a cluster of four real water molecules).

It is clear that the polarizable water beads attract each other stronger than the standard CG water beads because of additional electrostatic interactions between their charged particles WP and WM. This additional attraction should be counter-balanced by a reduced LJ self-interaction of W particles. Thus we tested less attractive interaction levels II, III and IV (the standard MARTINI water has the atom type P_4_, which has a self-interaction strength level I. Note that, for each of these levels, the LJ parameter σ_LJ_ = 0.47 nm. The LJ well depth ε_LJ_ = 5.0, 4.5, 4.0, 3.5 kJ mol^−1^ for levels I–IV, respectively). Concerning the LJ interactions between the W particles and other particles in the MARTINI force field, our expectation was that these could stay unaffected, i.e. correspond to those for a P_4_ particle (for the full interaction matrix, see [3]). However, as we will show below, the cross-interaction strength has to be reduced slightly in order to reproduce the correct partitioning behavior.

Since the polarization of water is treated explicitly in our polarizable model, the global dielectric constant *ε_r_* = 15 used in the standard MARTINI should be adjusted accordingly. This value of *ε_r_* compromises between large *ε* in water and small *ε* in the hydrophobic regions like the core of the lipid membrane. In the polarizable model, the global dielectric constant is reduced to *ε_r_* = 2.5 to ensure a realistic dielectric behavior in the hydrophobic regions. Other force field parameters are the same as in standard MARTINI [3].

### Simulation details

All simulations were performed with the GROMACS suite of programs, versions 3.3.1 [19], 4.0.2 and 4.0.5 [20]. Standard simulation parameters associated with the MARTINI force field [3] were used unless stated otherwise. A time step of 20 fs was used in all simulations. We have repeated some of the simulations using 10 fs and 30 fs time steps; the results were virtually identical to the ones reported below. Temperature and pressure were kept constant by using weak coupling schemes [21], with time constants of *τ_T_* = 0.3 ps and *τ_p_* = 3.0 ps, respectively. The distance *l* between the central W particle and the charged WP/WM particles was constrained using the LINCS algorithm [22]. Visualization of the results was done with VMD [23]. Error estimates were obtained using a block-averaging procedure [24]. Details of the system composition and set-up are given alongside the presentation of the results. Times are reported as actual simulation time, except when explicitly stated as effective time in order to compare the kinetics to either all-atom simulations or experiment. The effective time accounts for the speed-up in coarse-grained dynamics (see [4]) and equals four times the actual simulation time. The parameter files are available in Dataset S1. They can also be downloaded from http://cgmartini.nl, together with some example applications.

## Results

### Parameterization of the model

#### Particle density and dielectric properties of bulk water

To study the properties of bulk water, a system containing 400 polarizable water beads was simulated in a cubic box at NPT conditions, with *T* = 300 K and *P* = 1 bar. The charge *q* was changed from 0.38 to 0.50, the angle force constant *K_θ_* was varied between 0 and 6 kJ mol^−1^ rad^−2^, and the LJ self-interaction level was chosen either as level II, III, or IV. As explained in the Methods section, other parameters were kept fixed, the bond length *l* = 0.14 nm and the equilibrium angle θ = 0 rad. The systems were simulated for at least 150 ns at each of the parameters space points explored. We aimed to reproduce the experimental density and dielectric constant of bulk water. The density is straightforwardly obtained from the average box volume during the simulation. The dielectric constant *ε* is computed from the fluctuations of the total dipole moment *<M^2^>* of the system [25], using a Clausius–Mosotti-type equation [26]:
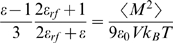
(1)where *ε*_0_ is the vacuum permittivity, *V* is the volume, *k_B_* is Boltzmann's constant, *T* is the temperature, and *ε_rf_* is the dielectric permittivity of the continuum if a reaction field correction is used. In this work, *ε* was computed as implemented in the g_dipoles program from the GROMACS suite, with *ε_r_* = 2.5, *ε_rf_* = ∞ (which is interpreted as the absence of the reaction field correction in GROMACS).

**Figure 2:**
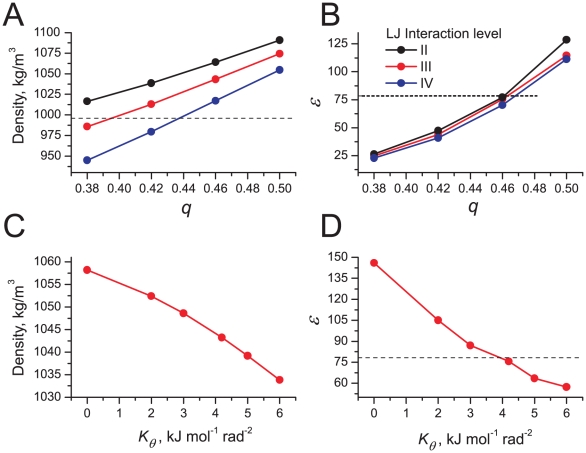
Particle density and dielectric constant of polarizable water at *T* = 300 K as a function of charge *q* and angle force constant *K_θ_*. In a) and b), *k* was kept fixed at 4.2 kJ mol^−1^ rad^−2^, whereas *q* was fixed to a value of 0.46 in c) and d). Dashed lines indicate the experimental dielectric constant (*ε* = 78.4 at 298 K) and the density of real water (ρ = 996 kg m^−3^ at 300 K [31]), respectively.

Fig. 2 shows the particle density and dielectric constant *ε* as a function of the charge *q* and the angle force constant *K_θ_*. The density increases linearly with *q* over the studied charge range (Fig. 2a). The LJ interaction level II yields a density that is above the experimental density along the studied range of charges (black curve). Reducing the interaction to level III results in a shift toward lower densities (red curve), with the target density being met at about *q* = 0.4. Decreasing the interaction strength even further yields the experimental density at about *q* = 0.45 (level IV, blue curve). Fig. 2b shows a strong dependence of the dielectric constant on the charge. For interaction levels II and III (black and red curves, respectively), a charge of 0.46 results in dielectric constants of 77.1 and 75.6, respectively, which are close to the experimental target value of 78.4 at 298 K (dashed line) [27]. For level IV interaction strength (blue curve), a slightly larger charge of about *q* = 0.47 is required to obtain the experimental dielectric constant.

From Fig. 2a and 2b, it seems that interaction level IV in conjunction with a charge of 0.47 would be an optimal choice, as such a model would yield the experimental dielectric constant at a density of about 1028 kg m^−3^, which is only about 3% too high. However, with level IV interaction strength the hydration free energy of water, and the related water/vapor surface tension were both found to become too low (−16.0 kJ mol^−1^ and 24.7 mN/m, respectively, for the level IV model, as compared to −18.7 kJ mol^−1^ and 30.5 mN/m for the level III model). Thus, we settled for the model with LJ interaction level III and charge 0.46, which yields a density that is 4.7% too large (1043 kg m^−3^).

Fig. 2c and 2d show the influence of the angle force constant *K_θ_* on the density and dielectric constant, respectively, obtained with the model with LJ interaction level III and q = 0.46. For the small force constants studied, the density drops moderately with increasing *K_θ_* (Fig. 2c). By contrast, the dielectric constant strongly depends on the angle force constant (Fig. 2d): *ε* = 75.6 is obtained by the optimal force constant of 4.2 kJ mol^−1^ rad^−2^, whereas in the absence of an angle potential (zero force constant) the dielectric constant increases to almost 150.

The final parameters of the polarizable MARTINI water model are summarized in Table 1, together with some selected properties which are detailed further below. The model bears charges of *q* = ±0.46 at the WP and WM particles, respectively, which are constrained at a fixed distance of 0.14 nm with respect to the central W particle (see Fig. 1). A harmonic angle potential with a force constant *K_θ_* = 4.2 kJ mol^−1^ rad^−2^ and an equilibrium angle θ = 0 degrees is added. The LJ interaction between the water beads is described by a level III interaction (LJ 12-6 potential with ε_LJ_ = 4.0 kJ mol^−1^ and σ = 0.47 nm).

**Table 1:** Final parameter set and selected properties of the polarizable water model.

Parameters		Properties^a^	
charge WP,WM	*q* = ±0.46	density	1043 kg m^−3^
bond W-WP, W-WM	*l* = 0.14 nm	dielectric constant	75.6
angle WP-W-WM	*θ* = 0 rad	dipole moment	4.9 Debye
	*K_θ_* = 4.2 kJ mol^−1^ rad^−2^	self diffusion	2.5 10^−5^ cm^2^ s^−1^
LJ_W-W_	*ε* = 4.0 kJ mol^−1^	hydration free energy	−18.7 kJ mol^−1^
	*σ* = 0.47 nm	freezing temperature	282±3 K
relative screening	*ε_r_* = 2.5	air/water surface tension	30.5 mN/m

a All properties obtained at *T* = 300 K.

#### Partitioning behavior

The MARTINI force field is based on the reproduction of partitioning free energies between water and organic solvents. With the new polarizable water model, the self-interactions of bulk water have been changed with respect to that of the standard MARTINI water model. Consequently, the partitioning free energies *ΔG^part^* of the CG particle types need to be re-evaluated. Since the solvation free energy *ΔG^solv^* of the CG particles in organic solvents has not changed (these interactions have not been affected, except for interactions involving charged Q type particles, see below), it suffices to calculate the hydration free energies *ΔG^hydr^*; the partitioning free energy is obtained from *ΔG^part^ = ΔG^hydr^−ΔG^solv^*. The hydration free energies of selected bead types were obtained by the thermodynamic integration (TI) method [28], decoupling the solute bead from its surrounding solvent molecules by turning all solute-solvent interactions off. Simulations were performed at 300 K. Twenty-two simulations, each of length 6 ns, were performed at evenly-spaced λ-values ranging from 0 to 1. A soft core potential was used for the non-bonded interactions. The derivative of the free energy with respect to λ, ∂*G*/∂*λ*, was integrated using the trapezoidal method.

Three different polarizable water models were tested. The first model uses exactly the same cross-interactions between the polar W particle and the other particles as for the standard W particle, i.e. the central particle is modeled as type P_4_. The other two models are obtained by adjusting the cross-interactions to either 95% or 92% of the strength defined for the P_4_ particle, except for interactions with the charged Q-type particles, which are treated differently (see below). This corresponds to a scaling of the LJ well-depth, i.e. *ε*_LJ_ = *a ε*_LJ_ with *a* = 0.92 or 0.95. In addition, the hydration free energy for the standard MARTINI model was calculated. Note that, in the standard MARTINI model, experimental hydration free energies are not reproduced. Although the correct trend is observed, the actual values are systematically too high. This is a known consequence of using a LJ 12-6 interaction potential, which has a limited fluid range. As long as the applications are aimed at studying the condensed phase, the most important thermodynamic property is the partitioning free energy. Our aim is therefore to reproduce the hydration free energy of the standard model.

The results of the free energy calculations are summarized in Table 2. We first point out that the TI results differ slightly from the values published in the original MARTINI paper [3]. This is presumably due to finite concentration effects affecting the original calculations, in which the hydration free energy was obtained from the direct partitioning of solute beads between water and vacuum. The TI results, which truly reflect infinite dilution, are considered more accurate. The statistical accuracy of the TI hydration free energies is within ±0.3 kJ mol^−1^. Comparing the three different polarizable water models shows that in the case of the 100% level model, the hydration free energies are too favorable. The other two models, both 92% and 95%, reproduce the hydration free energy of the standard MARTINI model to a better extent. Based on further tests described below, the 95% model was eventually chosen since it reproduces best the experimental behavior of lipid membranes. The hydration free energy of water itself, which does not depend on the cross-interactions, is −18.7 kJ mol^−1^, comparable to that of the standard model (cf. P_4_ hydration free energy, −18.5 kJ mol^−1^).

**Table 2:** Hydration free energies *ΔG^hydr^* for selected bead types, in kJ mol^−1^.

Bead Type	Standard MARTINI^a^		Polarizable MARTINI^b^		
	*Partitioning*	*TI*	*92%*	*95%*	*100%*
Q_da_	−25	−24.7	-	-	−73.8^d^
Q_d_/Q_a_	−25	−24.7	-	-	−67.5^d^
Q_0_	−25	−24.7	-	-	−62.8^d^
P_5_	−25	−24.7	−23.0	−24.5	−27.7
P_4_/P_3_	−18	−18.5	−17.0	−18.7	−21.8
P_2_/P_1_	−14	−13.6	−11.9	−13.5	−15.6
N_da_/N_d_/N_a_	−9	−7.8	−7.2	−8.3	−10.9
N_0_	−2	−2.8	−2.3	−3.2	−5.2
C_4_/C_3_	5	4.7	5.1	4.0	2.9
C_1_	14	11.6	11.1	10.6	9.7
POL^c^	-		-	-	−18.7

The statistical errors of *ΔG^hydr^* obtained via TI are below 0.3 kJ mol^−1^.

a ‘Partitioning’ values are based on partitioning between vapor and liquid water, obtained from ref [3]; TI values are calculated in the current manuscript using Thermodynamic Integration.

b Values obtained by TI reducing the interaction strength between polarizable water and other beads to 100%, 95%, and 92% of the standard interaction of a P_4_ particle type.

c Hydration free energy of the polarizable water model.

d Interaction level defined in Table 3.

The hydration free energy of charged particles (type Q) deserves a more elaborate discussion. In the original MARTINI model, the interaction of charged groups with water particles is of comparable strength to that of other polar groups. Hence, the hydration free energy of charged groups is also similar (−24.7 kJ mol^−1^). Even though charged groups, especially small ones like single ions, are considered as having an implicit hydration shell in their MARTINI representation, their hydration free energy is grossly underestimated. Consequently, ions were observed to partition into apolar media far too easily in the first version of the CG model [4]. In the next version of the model [3], coined MARTINI, a pragmatic solution was presented by increasing the effective size for ions entering apolar media. Pragmatic as it might be, it is an ad-hoc solution making little sense from a physics point of view. With the polarizable water model the hydration free energy of charged particles increases naturally, due to electrostatic interactions between the ionic charge and the polarizable charges embedded in the water model. Consequently, the interaction between Q type particles and C_1_/C_2_ type particles can be modeled with the normal range of interaction strengths also used for the other particles. However, test-simulations on binding of peptides to membranes (not shown) indicated that the hydration of charged groups is a little too strong, preventing binding of amphiphilic and hydrophobic peptides to the lipid/water interface. To compensate for the strong electrostatic contribution to the hydration free energy, the LJ interaction between Q type particles and the polarizable water particles is reduced, resulting in the free energies of around −70 kJ mol^−1^ as reported in Table 2. In addition, the relative interaction of Q type particles with other particle types has generally been increased, and their self-interaction decreased. The full list of changes of interactions involving Q type particles is given in Table 3. In the application section on the permeation of ions through lipid membranes, we will show that the new model results in more realistic behavior, without the need for unphysical fixes.

**Table 3:** Overview of interaction levels for charged particle types^a^.

	POL	Q_da_	Q_d_	Q_a_	Q_0_	P_5_	P_4_	P_3_	P_2_	P_1_	N_da_	N_d_	N_a_	N_0_	C_5_	C_4_	C_3_	C_2_	C_1_
Q_da_	**O**	**O**	**I**	**I**	**IV**	**O**	**O**	**O**	**O**	**O**	**O**	**O**	**O**	**III**	**IV**	**V**	**VI**	**VII**	**VII**
	O	O	O	O	II	O	O	O	I	I	I	I	I	IV	V	VI	VII	IX	IX
Q_d_	**I**	**I**	**IV**	**III**	**VII**	**O**	**O**	**O**	**O**	**O**	**O**	**II**	**O**	**III**	**IV**	**V**	**VI**	**VII**	**VII**
	O	O	I	O	II	O	O	O	I	I	I	III	I	IV	V	VI	VII	IX	IX
Q_a_	**I**	**I**	**III**	**IV**	**VII**	**O**	**O**	**O**	**O**	**O**	**O**	**O**	**II**	**III**	**IV**	**V**	**VI**	**VII**	**VII**
	O	O	O	I	II	O	O	O	I	I	I	I	III	IV	V	VI	VII	IX	IX
Q_0_	**II**	**IV**	**VII**	**VII**	**IV**	**O**	**O**	**O**	**I**	**II**	**II**	**II**	**II**	**III**	**IV**	**V**	**VI**	**VII**	**VII**
	O	II	II	II	IV	I	O	I	II	III	III	III	III	IV	V	VI	VII	IX	IX

a New values in bold font, old values underneath in normal font. Level of interaction indicates the well depth ε in the LJ potential: O, ε = 5.6 kJ/mol; I, ε = 5.0 kJ/mol; II, ε = 4.5 kJ/mol; III, ε = 4.0 kJ/mol; IV, ε = 3.5 kJ/mol; V, ε = 3.1 kJ/mol; VI, ε = 2.7 kJ/mol; VII, ε = 2.3 kJ/mol; VIII, ε = 2.0 kJ/mol; IX, ε = 2.0 kJ/mol. The LJ parameter σ = 0.47 nm for all interaction levels except level IX for which σ = 0.62 nm.

To illustrate the behavior of the original and polarizable water model with respect to partitioning, we calculated the free energy profile for a butane molecule (represented by a single bead of type C_1_) across a hexadecane/water interface. The system is composed of 3708 CG waters and 776 hexadecane molecules. Umbrella sampling simulations were used to compute the free energy profile of the butane bead as a function of the distance from the water-hexadecane interface. Umbrella sampling simulations were performed for 60 windows, with a restraining force constant of 500 kJ mol^−1^ nm^−2^. Each window was simulated for 25 ns.

**Figure 3:**
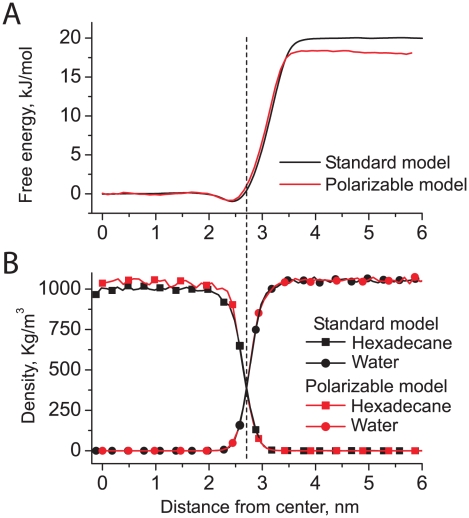
Properties of water-hexadecane interface. a) Potential of mean force for CG butane, and b) particle density profiles for the standard and the polarizable water models (black and red curves, respectively).

The potential of mean force is shown in Fig. 3 along with the distributions of the particle densities. It is clearly seen that the shape of the free energy profiles is quite similar for the standard and the polarizable water models (Fig. 3a). The distributions of particle densities are also almost identical (Fig. 3b). The free energy differences between the bulk phases are *ΔG* = 20 kJ mol^−1^ for standard MARTINI and *ΔG* = 18 kJ mol^−1^ for the polarizable model. As expected 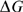 in polarizable water is slightly lower than in standard MARTINI water (see Table 2). Both values are in good agreement with the experimental value of *ΔG* = 18 kJ mol^−1^ [3].

### Testing of the model

#### Distribution of dipole moments

**Figure 4:**
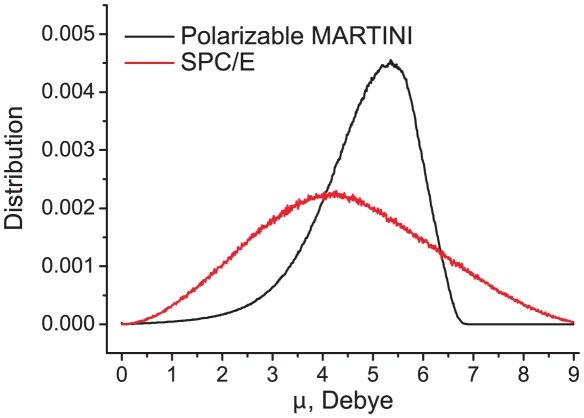
Distributions of the dipole moment in polarizable MARTINI water (black curve) and SPC/E water (red curve).

Fig. 4 compares the dipole moment distributions obtained for the polarizable MARTINI water model to the one obtained from a 10-ns simulation of 1600 SPC/E [29] water molecules. To enable the comparison, the dipole moment for SPC/E was calculated for groups of four, randomly chosen, molecules. The distribution obtained for SPC/E water is broad, with an average dipole moment of 4.4 Debye. The polarizable MARTINI water yields a sharper and more distinctly asymmetric distribution, with an average dipole moment of 4.9 Debye. During the parameterization stage, we tested several alternative parameter sets in order to improve the overlap, but there are a couple of opposing factors that limit the degree of matching one could achieve. The maximum dipole moment that can be obtained with the polarizable CG water model is given by 2*ql*, corresponding to 6.2 Debye for the current model. SPC/E water, however, has a maximum dipole achieved by perfect alignment of four water molecules with a dipole of 2.35 Debye each, amounting to 9.4 Debye. The only way to extend the range of accessible dipole moments for the CG model is to substantially increase either the charge *q* or length *l*. The latter option is not possible (the charged particles should remain well embedded within the LJ radius of the W particle), whereas the first option leads to an overshoot of the dielectric constant (cf. Fig. 2b). This could in principle be counteracted by a concomitant increase of the angle force constant *K_θ_* (cf. Fig. 2d), which however, would result in a distribution that is too sharp. Altogether, as our main aim is to generate a model that reproduces the global dielectric properties of experimental water as close as possible, the agreement between the distributions of the polarizable MARTINI water and SPC/E as shown in Fig. 4 is reasonable. One should also keep in mind that it is unclear to what extent the dipole moment distribution obtained with the atomistic model actually represents real water.

#### Temperature dependence of density and dielectric constant

**Figure 5:**
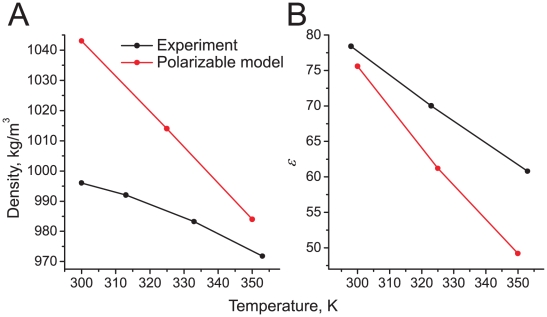
Temperature dependence of polarizable water model. Particle density (a) and dielectric constant (b) are compared to experimental values (taken from [27], [31]).

Next, we investigated the temperature dependence of the density and dielectric constant. Fig. 5 compares these properties obtained with the polarizable water model to experiment, covering a temperature range from 300 K to 350 K. The system is the same as the one used for the parameterization (see above). Starting from the value of 1043 kg m^−3^ at 300 K (cf. Table 1), the density-drop of the polarizable water model is more pronounced as compared to experiment (Fig. 5a). At 350 K, the density of the CG water is 984 kg m^−3^, only about 1% higher than in real water. Fig. 5b shows that, similar to the density, the dielectric constant of CG water depends slightly stronger on temperature compared to real water: whereas the measured *ε* drops from 78.4 at 298 K to 60.8 at 353 K, the *ε* of the CG water decreases from 75.6 at 300 K to 49.0 at 350 K. Taken together, the experimental trends of a decreasing density and dielectric constant with increasing temperature are well reproduced.

#### Diffusion constant

The self-diffusion coefficient of a polarizable water bead is *D_CG_* = 6.25·10^−6^ cm^2^ s^−1^ at 300 K, as calculated from the mean-square-displacement (msd) over effective time. As one CG bead represents four real water molecules, and the average msd of the center of mass of four molecules is four times smaller than the average of the individual msd's of these molecules, the effective diffusion coefficient of individual water molecules represented by a CG water bead is 4·*D_CG_* = 2.5·10^−5^ cm^2^ s^−1^. This value is slightly higher than the self-diffusion coefficient of the original MARTINI water model of 2·10^−5^ cm^2^ s^−1^ and compares well to the experimental diffusion coefficient of 2.3·10^−5^ cm^2^ s^−1^ at 300 K [30].

#### Surface tension of the water/vapor interface

To test whether or not the polarizable model behaves differently from the standard MARTINI water model near an interface, we evaluated the water/vapor surface tension. The system simulated contained 3708 water beads arranged into a slab in the XY plane; the water slab was surrounded by a vacuum slab in the Z direction. The system was simulated for 40 ns under NVT conditions at T = 300 K. The surface tension γ was computed as
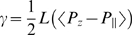
(2)where *P_z_* and *P_∥_* denote the perpendicular and the lateral pressure components, respectively, < > denotes an ensemble average, and *L* is the box length. Our polarizable water model yielded a water/vapor surface tension of 30.5 mN/m. This value correlates well with the values obtained for standard MARTINI force field (30–45 mN/m depending on the system size [3]). The size of the system used in this work corresponds to the “large” system used in the work [3] and should therefore be compared with the corresponding value of 30 mN/m. However, it is still quite far from the experimental value of 73 mN/m [31], [32], and in this respect the polarizable model does not improve on the non-polarizable version. Alignment of dipoles near the water/vapor interface is observed in atomistic water models [33], but there is no significant ordering of the dipoles of the CG water beads near the water-vacuum interface (data not shown). As we will show below, in the case of a water/lipid interface significant water ordering does take place.

#### Freezing point of water

To determine the freezing point of the polarizable water model, a system containing 1800 polarizable waters was simulated under constant pressure of 1 bar and at constant temperature in the range 270–300 K. The starting configuration consisted of a system in which liquid and frozen water coexists. Simulations were run for 100 ns. For temperatures of 285 K and above, the frozen water was observed to melt, whereas for temperatures of 280 K and below the complete system eventually crystallized. We conclude that the polarizable water model has a melting temperature 280 K <* T_melt _*< 285 K. Although the melting temperature is a bit higher than the experimental freezing point (273 K), it has improved with respect to the standard MARTINI model for which *T_melt_* = 290±5 K (determined using the same method [4]).

#### Radial distribution functions of ionic solutions

**Figure 6:**
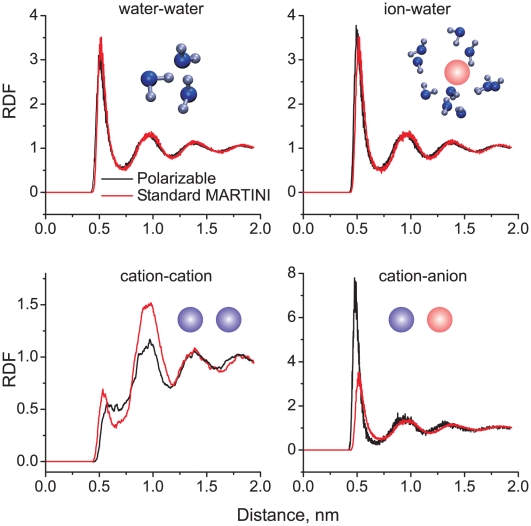
Radial distribution functions (RDFs) of ions and water in a 0.4 M NaCl salt solution. Insets show the molecules used to compute the RDFs.

In order to study the interaction of the polarizable water molecules with charged CG beads we performed simulations of a 0.4 M salt solution consisting of 485 CG waters, 16 CG chloride anions (Q_a_ type) and 16 CG sodium cations (Q_d_ type). A reference system of the same composition but containing non-polarizable MARTINI water beads was also simulated. Both systems were simulated for 200 ns at constant pressure of 1 bar and constant temperature of 325 K. The radial distribution functions (RDFs) of the pairs of particles of different type are shown in Fig. 6.

It is clearly seen that the RDFs of the water-water and water-ion pairs are almost identical in the standard MARTINI and in the polarizable model. This means that additional attraction between the water beads caused by the charged particles is counterbalanced almost precisely by the reduced attraction of W-particles. The positions of the peaks in the cation-cation (which is the same as anion-anion) and the cation-anion RDFs are almost identical in the polarizable model and in the standard model. The heights of the peaks, however, do not match exactly, especially in the case of the sodium-chloride interaction. In the polarizable CG model the sodium-chloride pair has an almost three fold higher occurrence probability compared to the standard model, corresponding to a relative stabilization of the contact pair of the order of *k_B_T*. Which of the two is more realistic is hard to tell; the original model was optimized with respect to a particular set of atomistic simulation data, however, large differences between atomistic force fields exist [34]. Keeping in mind that formation of the contact pair at low or moderate ionic strength has a low probability due to entropic reasons, the differences between the two models are likely of little importance for simulations at physiological conditions. Whether or not this is true for the properties of other charged groups such as the lipid head groups is investigated next.

#### Properties of a lipid bilayer

**Figure 7:**
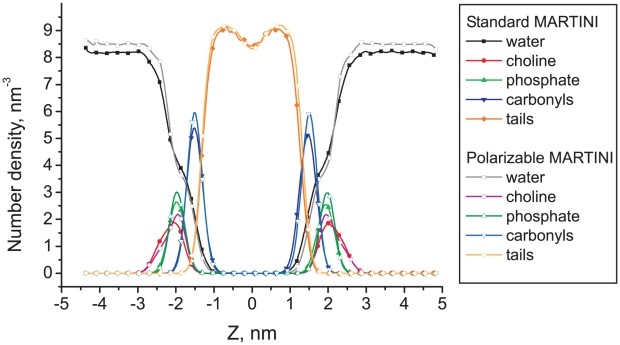
Distributions of the particle density for different CG groups of DPPC bilayer, with respect to the bilayer center (Z = 0).

A bilayer containing 128 DPPC (dipalmitoyl-phosphatidylcholine) lipids and 2000 CG waters was simulated, both with polarizable and non-polarizable water models. The simulations were performed at constant temperature of 323 K and constant pressure of 1 bar. Semi-isotropic pressure coupling was used, allowing the bilayer to be in a tensionless state. Both systems were simulated for 100 ns. The average area per lipid is found to be 0.644 nm^2^ in the case of non-polarizable water, and 0.635 nm^2^ in combination with the polarizable water. As shown in Fig. 7, the structure of the bilayers is almost identical in both simulations. The complex lipid/water interface, where polarization effects are expected to be significant, is structurally unaffected by the change in water model apart from the anticipated increase of the density of the bulk water phase. We conclude that the reparameterization of the water model did not change the structural properties of a lipid bilayer to a significant extent. Considering the general good agreement of bilayer properties for the MARTINI model in comparison to atomistic models and experiment [4], this is encouraging.

**Figure 8:**
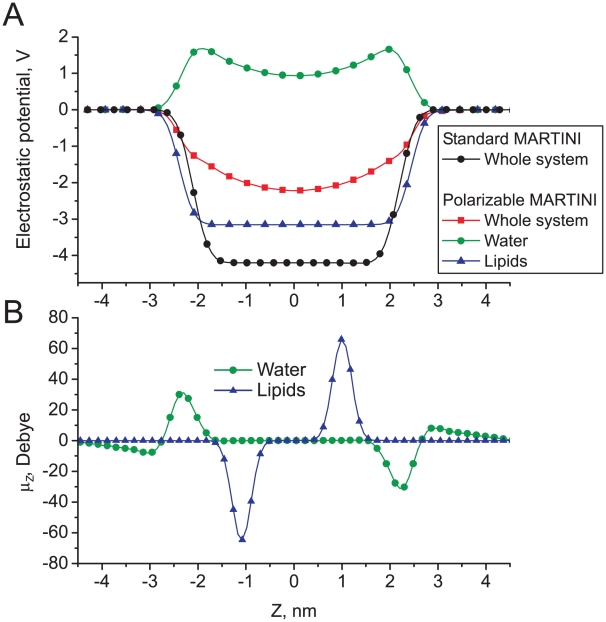
Polarization effects across a DPPC bilayer (bilayer center at Z = 0). a) Electrostatic potentials across the bilayer for both standard and polarizable water models. b) Distribution of the dipole moment across the bilayer in the case of polarizable water. Only the Z-component of the dipole moment is shown.

Whereas structurally the bilayer does not seem to respond much to the presence of a polarizable solvent, there is an important difference regarding the electrostatic potential across the bilayer. The electrostatic potential across the bilayer, obtained from a double integration of the local charge density, is shown in Fig. 8a for both models. The potential is split into contributions arising from water and lipids for the polarizable model. In the case of the standard model, the electrostatic potential across the system is created by the lipids only, since the water model lacks any charges. Due to the preferred orientation of the phosphate-choline head group dipole pointing slightly toward the solvent phase, a substantial negative electrostatic potential is created inside the membrane. In the case of polarizable model, the total electrostatic potential is a sum of contributions from both lipid head groups and water beads. The lipid contribution is similar in shape to the potential for the standard model, but its magnitude is smaller. However, the contribution from polarizable water is opposite, i.e. generating a positive potential inside the bilayer. The water therefore compensates for the lipid potential, reducing the overall potential difference across the bilayer from more than 4 V to less than 2 V. This is a direct and important consequence of the polarizable nature of the model. The sign of the total dipole potential is still at odds with results from AA simulations (e.g. [35], [36]), which predict that water actually is able to overcompensate the lipid potential resulting in an overall positive dipole potential inside the membrane.

To further characterize the behavior of the polarizable water beads at the lipid/water interface, we analyzed the distribution of the average dipole moment for both water and lipid head groups along the membrane normal (Z-axis). The result is shown in Fig. 8b. A clear orientation of the water dipoles at the interface can be appreciated, counter balancing the dipoles of the lipid head groups.

Finally, we studied the effect of the polarizable water on dynamical properties of the lipid bilayer by analyzing the lateral lipid diffusion. The self-diffusion constant, obtained from the slope of the mean-squared displacement of the lipids in the long time limit, is 1.1±0.1·10^−7^ cm^2^ s^−1^ with the new polarizable water model, as compared to 2.0±0.1·10^−7^ cm^2^ s^−1^ obtained with standard MARTINI water (effective time; statistical errors from separate analysis of the two individual monolayers). Thus, we conclude that the lateral diffusion is slowed down by almost a factor two with the new water model, due to electrostatic friction between the lipid headgroups and the water dipoles. Note, both values agree with experimentally determined diffusion coefficients, which are typically reported to be around 1·10^−7^ cm^2^ s^−1^ at temperatures close to 323 K [37], [38].

#### Effect of PME

The essence of CG models in general is to use short-ranged potentials in order to be computationally efficient. This choice is motivated by the fact that most of the interactions that drive self-organization in biomolecular systems are short-ranged. This is the case for e.g. hydrogen-bonding, steric repulsion, dispersion interactions, and also for collective effects such as hydrophobic interactions. Electrostatic interactions, however, are long-ranged. Similar to the approach used in AA simulations, one could include this long-range effect through the use of lattice sums as in PME [39]. The inclusion of long-range effects in simulations using the standard MARTINI model has already been shown to provide a more realistic description of the interaction of charged molecules with lipid membranes, e.g. in the case of dendrimers [40] and antimicrobial peptides [41].

With the new polarizable water model, the long-range effects are expected to be even more realistic considering the explicit screening. To test the effect of including long-range electrostatics on the equilibrium properties of bulk water as well as lipid membranes, we have recalculated the density and dielectric properties of water as well as the area per lipid of a DPPC lipid bilayer (see above) using PME electrostatics instead of shifted cut-off. A real-space cut-off of 1.2 nm, and a 0.12 nm Fourier grid spacing were used for PME. The use of PME results in about 0.6–1.1% lower densities as compared to shifted cut-off, with a similar temperature-dependence. In line with the lower density, the diffusion rate is slightly faster (2.5, 3.7, and 4.7 10^−5^ cm^2^ s^−1^ with PME versus 2.5, 3.2, and 4.4 10^−5^ cm^2^ s^−1^ without PME, at 300, 325, and 350 K, respectively). Furthermore, PME yields a slightly higher dielectric constant for temperatures below 350 K and a stronger temperature-dependence: *ε* drops from 82.4 at 300 K to 49.2 at 350 K. Finally, the area per lipid for the DPPC bilayer is 0.648 nm^2^ with PME, which can be compared to the 0.635 nm^2^ obtained with shifted cut-off (see above). Thus, PME and shifted cut-off yield very similar results, and we conclude that the polarizable MARTINI water model can be used equally well with both settings.

### Application of the model

#### Translocation of ions through a lipid bilayer

A realistic description of the partitioning of charged groups into a medium of low polarity has always been a weak spot of standard CG models. With the polarizable water model we expect that applications involving charge transfer are more realistic. Here we describe the translocation of Na^+^ and Cl^−^ CG ions through a DPPC bilayer, through computation of the potential of mean force (PMF). The system set-up is the same as described above. To construct the PMF, umbrella sampling was used constraining the ion to different distances from the center of the bilayer. A total of 80 umbrella sampling windows were used, with a restraining force constant of 400 kJ mol^−1^ nm^−2^. Each window was simulated for 25 ns. Simulations were also performed with PME electrostatics, as well as with the standard water model for reference. As it was mentioned above, the hydration free energy of the ions was grossly underestimated in the early version of MARTINI force field [4]. In this version the Q_a_/Q_d_−C_1_ interactions were set to level VIII, while the level IX was used for these interactions in the later version [3]. The PMFs for level VIII parameters (referred as “early MARTINI”) were also computed for the sake of comparison.

**Figure 9:**
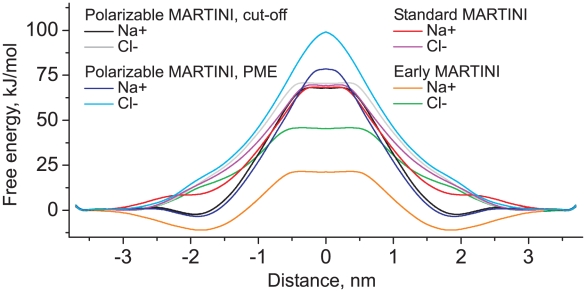
PMFs of translocation of Na^+^ and Cl^−^ ions through a DPPC bilayer, with respect to the distance from the membrane center. The standard water model is compared to the polarizable water model with and without long-range electrostatics (PME).

The potentials of mean force (PMFs) are shown in Fig. 9, and the corresponding barrier heights are summarized in Table 4. It is clearly seen that the PMFs for Na^+^ and Cl^−^ are quite similar in the case of the standard model. The free energy barrier for Cl^−^ is only marginally higher than for Na^+^. In the case of polarizable water with shifted cut-off electrostatics, the heights of the barriers for both Na^+^ and Cl^−^ are very similar to those of the standard model. However, the shapes of the PMFs are different. The width of the central barrier for Cl^−^ is larger then in the standard model. There is also a pronounced energy well for Na^+^ ions in the region of the lipid head groups, which indicates strong binding of Na^+^ with the head groups. No such binding is observed for Cl^−^ ions. Preferred binding of sodium, to the carbonyl groups, is also observed in atomistic simulations [42], [43].

**Table 4:** Heights of the energy barriers (in kJ mol^−1^) for Na^+^ and Cl^−^ translocation across a DPPC membrane.

	Standard (*cut-off* )	Early (*cut-off*)	Polarizable (*cut-off*)	Polarizable (*PME*)	Atomistic^a^ (*PME*)
Na^+^	68.0	21.0	67.6	78.6	91.7
Cl^−^	69.2	45.3	70.4	99.0	98.8

Different methods are compared, either using the standard MARTINI, early MARTINI or the current polarizable water model, and either with cut-off or long-range electrostatics (PME).

a Atomistic data for DMPC, taken from [45].

In the case of PME electrostatics the long-range interactions lead to some changes in the PMFs, especially in the central membrane region. The energy barriers are systematically higher for both types of ions. The barrier for Cl^−^ increases even substantially, which makes it ∼20 kJ mol^−1^ higher than the barrier for Na^+^ in the case of PME. The overall increase of the barrier height with PME has previously been observed in MD simulations, and is explained by the interaction between the periodic images of the system in the lateral plane [44]. It is therefore an artifact of PME. The relative increase of the Cl^−^ barrier with respect to the Na^+^ barrier, however, might be a realistic long-range effect as it is also probed in atomistic simulations (see below). Note also the rounding of the top of the barrier in the case of PME, reflecting the parabolic shape of the electrostatic potential (cf. Fig. 8a).

The PMFs for early MARTINI differ dramatically from the PMFs for other models. The heights of the barriers are underestimated severely for both ions and the difference between the heights for Na^+^ and Cl^−^ is too large. In contrast, the binding of Na^+^ with the head groups is overestimated significantly. It is interesting that the standard MARTINI shows realistic barrier heights, but lacks the binding of Na^+^ with the lipid heads, while the early MARTINI shows such binding, but leads to wrong barrier heights. The polarizable model allows to describe the binding of Na^+^ correctly alongside with realistic barrier heights.

It is also of interest to compare our data with the PMFs for translocation of Na^+^ and Cl^−^ ions through a DMPC bilayer obtained recently in atomistic MD simulations [45]. The shapes of the PMFs are in very good correspondence for both ions (see Fig. 9 and Fig. 2 in [45]). The atomistic PMFs exhibit a pronounced energy well in the head group region for Na^+^, which is also observed in our polarizable model, but not in the standard model. The heights of the barriers are compared in Table 4. The height of the barrier for Cl^−^ is almost identical in the polarizable model with PME compared to the atomistic model, which was also simulated with PME. The barrier for Na^+^ is somewhat underestimated in the polarizable model in comparison to atomistic results, but still shows significant improvement in comparison to the non-polarizable model. Both models predict a substantial larger barrier height for Cl^−^ versus Na^+^, which has been attributed [45] to the specific interactions of the Na^+^ ion with the phosphate and carbonyl groups facilitating the translocation of the cation. Note that the nature of our CG model does not allow us to discriminate between lipid tail lengths differing in only one or two methylene groups. Our model for DPPC also models DMPC, and hence, the comparison between our CG results and the atomistic results is meaningful and can not be improved by switching to another CG representation. Besides, our main point here is to compare barrier heights. A (slightly) different membrane thickness clearly changes the width of the barrier, but not so much its height.

#### Electroporation

Experimentally, membranes can be porated by a process called electroporation, in which an external potential difference is applied to a membrane or cell. Voltage differences across a membrane due to application of nanosecond length pulses have been estimated at up to several volts. This setup can be mimicked in atomistic simulations of membranes by applying a constant electric field in the system [46], [47], [48], [49]. Here we tested two different electroporation events, namely the poration of an octane slab (as a simple mimetic of a lipid bilayer) by an external electric field, and the poration of a lipid bilayer due to the electric field created by an ionic imbalance across the membrane.

**Figure 10:**
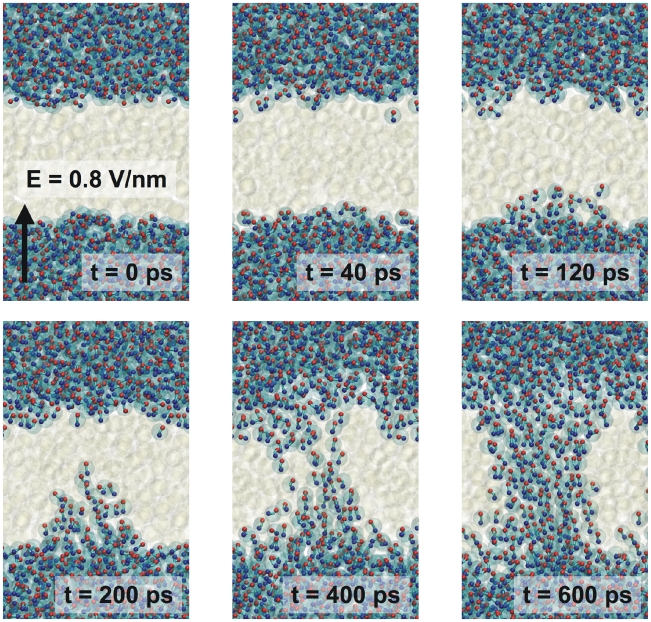
Electroporation of an octane slab by an electric field. Water is shown as balls and sticks. The octane slab is depicted as white transparent spheres. The direction and magnitude of the external field is indicated by the arrow.

Electroporation of an octane slab in water was studied using a setup very similar to the setup used in simulations performed by Tieleman using an atomistic model consisting of 182 octane and 1802 water molecules [50]. Our CG system also contained 182 octane molecules arranged as a slab in the XY plane surrounded by 450 polarizable water beads. The system was simulated in the NP_z_AT ensemble, with the pressure in the Z-direction coupled to 1 bar and the area fixed at 4.0×4.0 nm. The temperature was 300 K. The electric field was applied in the Z direction. Field strengths of 0.4–0.8 V nm^−1^ were used, and simulations were run for 400 ns effective time. We found that fields weaker than 0.5 V nm^−1^ did not induce any significant changes in the system on this time scale. However, fields of 0.5 V nm^−1^ and higher lead to electroporation of the octane slab. The higher the field, the faster the process, ranging from 10s of nanoseconds at E = 0.5 V nm^−1^ to sub-nanosecond poration at E = 0.8 V nm^−1^. In Fig. 10, snapshots are shown of the poration process observed in our simulations at high field strength. Starting from a small water finger protruding into the octane phase, a full water pore opens up in ∼500 ps. The pore keeps growing and, eventually, the octane slab becomes oriented parallel to the external field. A very similar mechanism is observed in the AA simulations reported by Tieleman [50], showing reproducible pore formation with a field of 0.8 V nm^−1^, on a time scale varying between 50 ps and 2 ns. A field of 0.5 V nm^−1^ or lower did not result in pore formation on these time scales. Thus, both the field strength required to initiate pore formation, and the kinetics of the process are in remarkable good agreement between the two models.

For the electroporation of a lipid membrane we mimicked the setup used in an atomistic study of Gurtavenko and Vattulainen [51]. The authors showed that a 128 lipid DMPC bilayer can be porated by a transmembrane ionic charge imbalance. An electric field of ∼0.4 V nm^−1^, created by an excess of 12 sodium ions on one side of the bilayer, caused the opening of a water pore on a nanosecond time scale. Our CG membrane system contained 512 DPPC lipids and 5640 polarizable waters, arranged in a double bilayer setup similar to that of the atomistic study [51]. In one of the water compartments, 52 sodium ions were placed. An equivalent amount of chloride ions were also added to neutralize the system, with the important notion that the chloride ions were distributed equally between the two water compartments. This set-up results in an ionic charge imbalance of 26e per bilayer, creating a field of 0.7 V nm^−1^ averaged across the system (evaluated from the integration of the charge distribution). Simulations were performed in the NP_Z_P_XY_T ensemble, with the pressure in the normal (Z) direction and lateral (X,Y) directions coupled independently to 1 bar. The temperature was maintained at 325 K, and multiple simulations were run for 400 ns effectively. Long-range electrostatics were included through PME.

**Figure 11:**
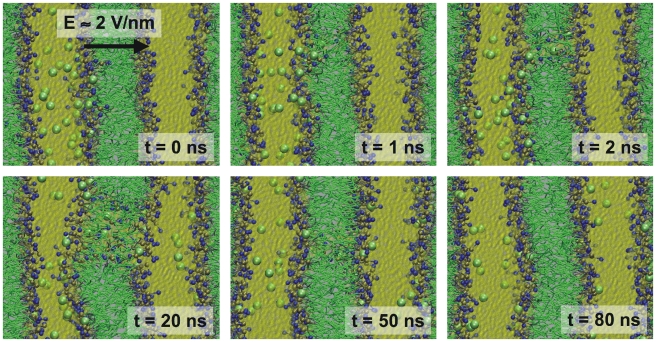
Electroporation of a DPPC membrane by an ionic imbalance. The polarizable water is shown as transparent yellow spheres, the lipid head groups as blue (choline) and golden (phosphate) spheres, the lipid tails as green sticks, and the sodium ions as large green balls. Formation of the pore is indicated by the yellow arrow. The direction and magnitude of the effective field is indicated by the black arrow.

The sequence of events of a typical simulation is shown in Fig. 11. Starting from two intact membranes separating two water compartments with an unequal amount of sodium ions (t = 0 ns), a small water pore is rapidly formed on a nanosecond time scale (t = 1 ns). The pore starts to conduct ions (t = 2 ns), discharging the ionic imbalance. Both sodium ions and chloride ions are transported, in opposite directions. The size of the pore grows till it reaches a maximum size of around 4 nm in diameter (t = 20 ns), after which the pore slowly closes again as the remaining electric field is becoming too small (t = 50 ns). After pore closure, ions can no longer permeate the membrane (t = 80 ns). The final ion distribution is 40 Na^+^/37 Cl^−^ ions in the compartment that originally contained all the sodium ions, and 12 Na^+^/15 Cl^−^ ions in the other compartment. These numbers imply that 12 Na^+^ ions have moved down the sodium gradient, with 11 Cl^−^ ions moving in the opposite direction. The remaining ionic imbalance is reduced to 3e, and the remaining field is discharged to 0.04 V nm^−1^. Repeated simulations, with randomly assigned initial velocities, show the same behavior on very similar time scales. Pores always open up within a few nanoseconds, and pore closure is observed in the range 10–60 ns. Occasionally, two pores are formed, however, always in the same membrane. The sequence of events, as well as the kinetics of the process, are in good agreement with those observed in the atomistic simulations [51]. Simulations in which a standard cut-off scheme was used instead of PME did not result in pore formation. This is no surprise considering that a distance of 1.2 nm (the cut-off radius) is not enough for the ions to actually feel the ionic imbalance across the membrane.

**Figure 12:**
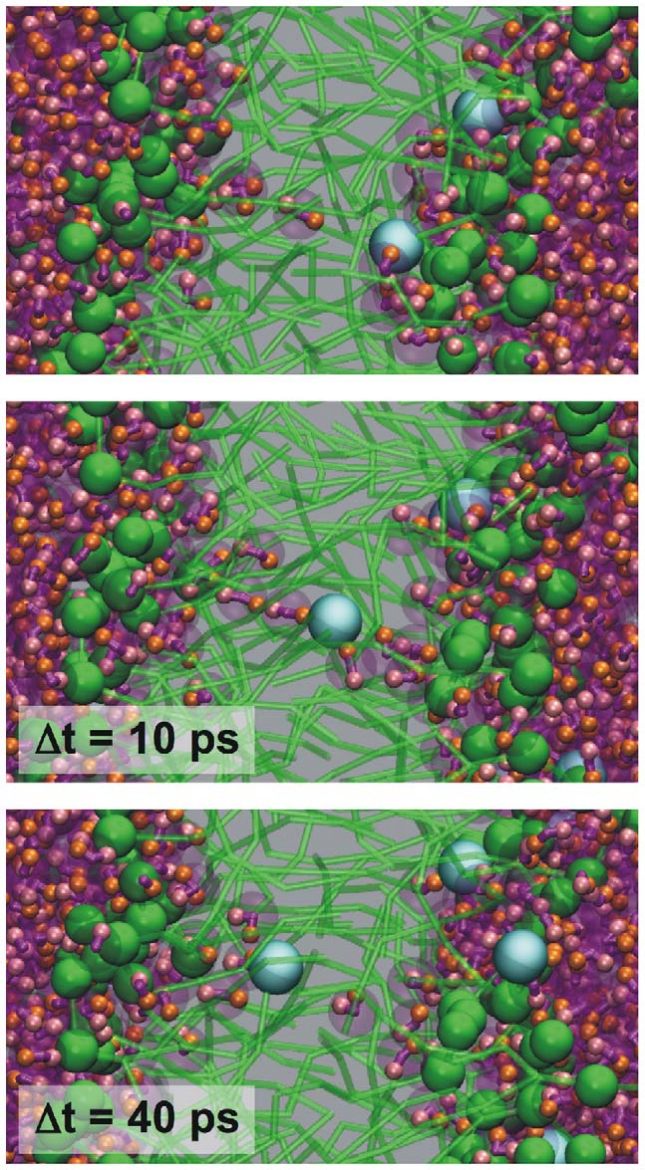
Ion leakage across a DPPC bilayer. Lipids are shown in green (heads as spheres, tails as bonds), sodium ions in cyan, and the polarizable water as purple, transparent beads with the positive WP particle in pink and the negative WM particle in orange.

We also studied the electroporation process at smaller field strengths 0.3–0.7 V nm^−1^, and found that pores are still spontaneously formed. The pore formation time, however, increases with decreasing field strength and may require >100 ns in the case of a system with an ionic imbalance of 10e (0.3 V nm^−1^). Independent of the initial field strength, we observed pore closure as soon as the field has been reduced to ∼0.04 V nm^−1^, corresponding to a remaining ionic imbalance of 3-4e. With an initial ionic imbalance of 6e (0.2 V nm^−1^), however, spontaneous pore formation is not observed even on a microsecond time scale. Interestingly, at intermediate field strengths we occasionally observed the leakage of single ions through the membrane without the formation of a pore. A sequence of such an event is shown in Fig. 12. Formation of a large water defect (so-called water finger [52], [53]), is seen to trigger the translocation process, which ends with the connection of two finger-tips from both sides of the membrane. The ion remains hydrated throughout the translocation process. Only Na^+^ ions were seen to cross the membrane in this manner.

## Discussion

The neglect of orientational polarizability in many water models associated with CG lipid force fields [54], [55], [56], [57], [58] is arguably one of the crudest approximations made. Water in those force fields is represented by spherically symmetric interaction sites either based on analytic potentials or effective potentials derived from atomistic simulations. None of these water models include electrostatic interactions, implying they are non-polarizable. The MARTINI model suffers from the same approximation. The inability to form a transmembrane water pore upon dragging a lipid across the membrane [3], or upon binding of antimicrobial peptides [41], [59], [60] are examples pointing at the shortcoming of the standard MARTINI water model.

To improve the behavior of the water model, inclusion of electrostatic interactions is needed; to account for the orientational polarizability, the minimum requirement is a point dipole, as in the models of e.g. Warshel and coworkers [9], [10], [13] and Orsi et al [61]. Our new water model is a three-bead model, consisting of a central particle with two charges-on-a-spring embedded, and was chosen as it combines simplicity with versatility. It is similar to the classical Drude model used in polarizable all-atom (AA) force fields to mimic electronic polarization [15], [16]. In contrast to the AA case, were the charged particles are massless and their position is solved in an expensive, self-consistent way, in our CG model the particles carry mass and follow the normal equation of motions. The model has only few adjustable parameters, yet enough of them to reproduce the dielectric properties of bulk water on the one hand and keeping at par with the standard MARTINI philosophy on the other. Despite the limited amount of free parameters in the model, a full exploration of parameter space is practically impossible; guided partly by intuition and partly through extensive testing we eventually settled on a combination of parameters which, overall, perform very well. Compared to the standard MARTINI water model, the polarizable model has improved properties, not only with respect to its dielectric behavior, but also for instance in the somewhat reduced freezing point. It can not be excluded that other combinations of parameters might perform even better, and we anticipate that further optimization of the model will take place in the future alongside with extending the range of applications of the model.

The main reason for having included polarizability into the model is the expectation that processes involving interactions between charged and polar groups in a low-dielectric medium are more realistically described. As an example we presented two applications for which standard CG models, including MARTINI, are less well suited, namely the translocation of ions across a lipid membrane and the electroporation of an octane slab and a lipid bilayer. Both processes involve the movement of charges from a high dielectric environment (water) to a low dielectric medium (membrane interior). A realistic description of such processes requires a model capable of performing local electrostatic screening. The two applications presented show that, despite being coarse-grained, our polarizable water model can do this at a level comparable to that of atomistic simulations. This opens the way to explore a number of important (bio)physical processes using the MARTINI model, including membrane poration by antimicrobial and cell penetrating peptides, DNA transfection, salt-induced membrane fusion, functioning of the voltage gated membrane channels, electroporation, and electrokinetic phenomena in general.

Finally, it is important to point out a few limitations of the polarizable water model: First, it is slightly more expensive from a computational point of view (for a pure water system the simulations are slowed down by a factor of approximately three). Second, the current parameterization of the model is not as thoroughly tested yet in comparison to the standard MARTINI model. For example lipid phase behavior, or the effect on proteins and peptides is largely unexplored. Third, despite an overall improved performance, some properties are still not at par with experimental measurements or data from atomistic simulations. These include the air/water surface tension, which is significantly too low, and also the sign of the membrane dipole potential which is opposite to that observed with more detailed force fields. Further improvement could be obtained by changing the analytical form of the non-bonded potential (i.e. moving away from the LJ 12-6 form), and by adding polarizability to other beads in the force field. The latter idea may also lead to a more realistic description of the protein backbone, allowing secondary structure formation to be described with MARTINI, an option we are currently exploring. We finally note that the polarizable MARTINI water model is not meant to replace the standard MARTINI water model, but should be viewed as an alternative with improved properties in some, but similar behavior at reduced efficiency in other applications.

## Supporting Information

Dataset S1 MARTINI topology and MD-parameter files for GROMACS.
(0.06 MB TAR)
